# Structure-based identification of SARS-CoV-2 main protease inhibitors from anti-viral specific chemical libraries: an exhaustive computational screening approach

**DOI:** 10.1007/s11030-021-10214-6

**Published:** 2021-04-12

**Authors:** Shovonlal Bhowmick, Achintya Saha, Sameh Mohamed Osman, Fatmah Ali Alasmary, Tahani Mazyad Almutairi, Md Ataul Islam

**Affiliations:** 1 Department of Chemical Technology, University of Calcutta, 92, A.P.C. Road, Kolkata- 700009, India; 2 Chemistry Department, College of Science, King Saud University, P.O. Box 2455, Riyadh, 11451 Saudi Arabia; 3 Division of Pharmacy and Optometry, School of Health Sciences, Faculty of Biology, Medicine and Health, University of Manchester, Oxford Road, Manchester, M13 9PL UK; 4 Department of Chemical Pathology, Faculty of Health Sciences, University of Pretoria and National Health Laboratory Service Tshwane Academic Division, Pretoria, South Africa

**Keywords:** SARS-CoV-2, Main protease, Molecular docking, Virtual screening, Molecular dynamics, MM-GBSA

## Abstract

**Abstract:**

Worldwide coronavirus disease 2019 (COVID-19) outbreak is still threatening global health since its outbreak first reported in the late 2019. The causative novel virus has been designated as severe acute respiratory syndrome coronavirus 2 (SARS-CoV-2). Although COVID-19 emergent with significant mortality, there is no availability of definite treatment measures. It is now extremely desirable to identify potential chemical entities against SARS-CoV-2 for the treatment of COVID-19. In the present study, a state-of-art virtual screening protocol was implemented on three anti-viral specific chemical libraries against SARS-CoV-2 main protease (M^pro^). Particularly, viewing the large-scale biological role of M^pro^ in the viral replication process it has been considered as a prospective anti-viral drug target. Herein, on collected 79,892 compounds, hierarchical multistep docking followed by relative binding free energy estimation has been performed. Thereafter, implying a user-defined XP-dock and MM-GBSA cut-off scores as −8.00 and −45.00 kcal/mol, chemical space has been further reduced. Exhaustive molecular binding interactions analyses and various pharmacokinetics profiles assessment suggested four compounds (ChemDiv_D658-0159, ChemDiv_F431-0433, Enamine_Z3019991843 and Asinex_LAS_51389260) as potent inhibitors/modulators of SARS-CoV-2 M^pro^. In-depth protein–ligand interactions stability in the dynamic state has been evaluated by 100 ns molecular dynamics (MD) simulation studies along with MM-GBSA-based binding free energy estimations of entire simulation trajectories that have revealed strong binding affinity of all identified compounds towards M^pro^. Hence, all four identified compounds might be considered as promising candidates for future drug development specifically targeting the SARS-CoV-2 M^pro^; however, they also need experimental assessment for a better understanding of molecular interaction mechanisms.

**Graphic abstract:**

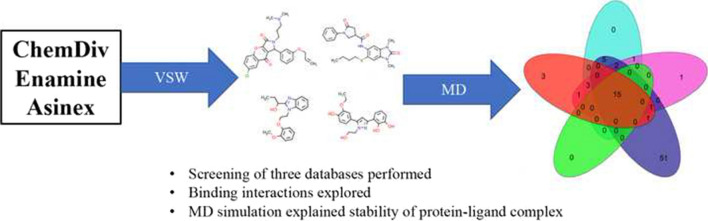

## Introduction

The coronavirus disease 2019 (COVID-19) has emerged as a very important public health concern since its outbreak was first reported at the end of December 2019, somewhere in Wuhan city in Hubei Province of China [1]. Initially, the causative agent of the COVID-19 outbreak was believed to be unknown [2]; however, later based on the report from several independent laboratories, the responsible agent identified as a severe acute respiratory syndrome—coronavirus 2 (SARS-CoV-2) also known as novel 2019-nCoV [3–5]. Evaluating the severity in spreading of COVID-19 and the contagious nature of infections, on January 30, 2020, World Health Organization (WHO) officially stated the COVID-19 situation as an epidemic and also announced such occurrence as “Public Health Emergency of International Concern” [6]. Afterward, on March 11, 2020, the Director-General of World Health Organization (WHO) declared the COVID-19 situation as a “pandemic” on the basis of “alarming levels of spread and severity, and inaction” [7]. However, till now no effective chemical entity has been identified or developed for curing or managing the emerging high-threat pathogen SARS-CoV-2. As per “Weekly Operational Update on COVID-19” on March 16, 2021, the explosive pandemic outbreak of COVID-19 reported 119, 791, 453 confirmed cases and 2,652,966 confirmed deaths. Such a scenario has explained the steadiness and persistent threat of this respiratory tract infectious disease to global health security as the infection rate still remains active almost after 1 year passed by since its outbreak. Hence, as of now maintaining social distancing is only the mainstay of COVID-19 management by means of preventing the spread of severe SARS-CoV-2 infection through respiratory droplets.

Ongoing researches have provided structural information of key proteins of SARS-CoV-2 and the other infected host as well that increases the possibility for the employment of structure-based drug design approach as the most promising strategy for COVID-19 therapeutics development [8]. Among many structurally well-characterized key proteins of SARS-CoV-2, the so-called main protease (M^pro^) (also known as 3C-like protease or 3CL^pro^)–a proteolytic enzyme has gained extremely important attention to the research communities for future drug development against COVID-19 [9–13]. Impeding the essential biological role of M^pro^ by small molecule or peptidomimetic inhibitor or peptide substrate has proved to be one of the most potential scientific basis for chemotherapeutics exploration to combat the COVID-19 pandemic [9, 12–16]. Particularly, blocking or modulating the SARS-CoV-2 M^pro^ protein activity subsequently may act as the inhibition of viral entry and reduced viral infectivity to the host cell [17]. SARS-CoV-2 encodes two proteolytic enzymes or cysteine proteases namely M^pro^ and papain-like cysteine protease (PL^pro^), and each enzyme specifically catalyzes the maturation or cleavage events of two overlapping polyproteins (replicase polyproteins 1a and 1ab) those are actually translated from the corona viral RNA genome [13, 14, 16, 18], which are necessarily mediate most of the biological functions required for the corona viral replication processes. As the key proteolytic enzyme, the M^pro^ specifically cleaves both the polyproteins to release a set of functional non-structural proteins, viz. nsp4–nsp16 [13, 14, 16, 18]. Specifically, M^pro^ can cleave or digest at least 11 conserved sites within large viral polyproteins [13, 14, 16, 18]. So, viewing the essential biological functions such as proteolytic processing of the polyproteins by hydrolysis and subsequent associations in the viral life cycle, SARS-CoV-2 M^pro^ is accounted as the most attractive and best-characterized drug targets among all proteins in coronaviruses [11–13, 18]. Another important factor for considering the M^pro^ protein as most the attractive drug target also lies in its high level of sequence conservation among other coronaviruses. Besides these two considerable facts, another reason for opting the SARS-CoV-2 M^pro^ protein for structure-based drug design strategy against COVID-19 therapeutic development is that no human proteases were found to pose similar substrate specificity like M^pro^ protein of SARS-CoV-2, therefore targeting the M^pro^ protein of SARS-CoV-2 for development of potential therapeutic against COVID-19 undoubtedly counted as a robustly significant approach [8].

The SARS-CoV-2 M^pro^ protein consists of three functional domains, viz. domain I extending from amino acid residues 10–99, domain II extending from amino acid residues 100 to 182, and domain III extending from amino residues 198 to 303. Domains I and II hold to be as an antiparallel β-barrel like structure, and the active site of SARS-CoV-2 M^pro^ protein is located in the cleft between these two domains [13, 16]. The SARS-CoV-2 M^pro^ protein contains two catalytic residues histidine/cysteine (His41 and Cys145) or catalytic dyad and also some binding pockets denoted as P1, P1′, P2, P3, and P4 [14]. Precisely, during the first step of the hydrolysis reaction residue Cys145 acts as the nucleophile; such reaction is majorly assisted by another residue His41 that acts as a base catalyst [16]. Alternately, the substrate binding pockets or sites (S1′-S1-S2-S4) of SARS-CoV-2 M^pro^ protein composed a number of amino acid residues, such as His41, Ser46, Met49,Tyr54, Phe140, Leu141, Asn142, Glu143, Cys145, His163, His164, Met165, Glu166, Leu167, His172, Phe185, Asp187, Gln189, Tyr190, Ala191, and Gln192 [8]. On the other hand, domain III owns as a globular cluster of five helices [10, 14–16] and is primarily associated with regulation of the dimerization of the M^pro^ protein by the formation of salt-bridge interaction between amino acid Glu290 of one protomer and another residue Arg4 of the other protomer of M^pro^ protein [13]. Moreover, the oxyanion loop extends from residues 138 to 146 of SARS-CoV-2 M^pro^ protein formed by the backbone amido groups of two amino acid residues Gly143 and Cys145 [16].

Herein, in the present study, employing a set of highly exhaustive computational methods comprising multistep molecular docking, long-range 100 ns molecular dynamics simulations studies, MM-GBSA-based binding free energy estimation of small molecules, and pharmacokinetics profile assessment have facilitated to identify of four compounds as potent inhibitors/modulators of SARS-CoV-2 M^pro^ protein. The outcomes of the present study provide valuable insights into the potential interaction mechanism upon binding of four small molecules inside the active catalytic site of SARS-CoV-2 M^pro^ that can direct the future structure-based drug design against COVID-19, specifically for highly selective potent inhibitors development for SARS-CoV-2 M^pro^. Nevertheless, searched out potential compounds also may need further optimization for exhibiting much better interaction mechanism and hence inhibition or modulation of the SARS-CoV-2 M^pro^ can be achieved.

## Materials and methods

Virtual screening (VS) is a computational approach to retrieve therapeutically effective molecules for a specific biomolecular receptor/protein target against any chemical database. It is increasingly being used by researchers in academia and pharmaceutical industries across the globe in order to tactically expediting the hit identification and lead optimization processes [19]. VS has become a very popular and effective approach due to its capability to screen out millions to billions of small molecules in a short time that minimize the timeline as well as the cost of the drug discovery crusade. The molecular docking-based VS is one of the widely used SBVS strategies in which the active binding mode and binding affinity of the molecules towards the target are estimated [20, 21]. The current study has been considered to screen three anti-viral specific chemical library databases through the multistep molecular docking followed by binding free energy estimation, *in silico* ADME and toxicity evaluation, and binding interactions stability assessment through MD simulation studies.

### Ligand and protein preparation

A pool of 79,892 small molecules was collected in 2D structural data format (sdf) from three databases: ChemDiv anti-viral (www.chemdiv.com), Enamine anti-viral (www.enamine.net) and Asinex anti-viral (http://www.asinex.com) databases. The entire set of molecules was prepared using the LigPrep module [22] of Schrödinger suite. After successful execution of LigPrep module, three-dimensional (3D) coordinates of the molecules were generated. Hydrogen atoms and appropriate charges were added to the molecules and followed by bad valencies corrected where required. The protonation and tautomeric states at pH 7.2 ± 2.0 of the molecules were generated using the Epik tool [23] implying OPLS forcefield [24]. Finally, for each molecule, the low-energy stereoisomers were developed and kept all molecules aside until considered for VSW protocol.

The 3D coordinates of the SARS-CoV-2 M^pro^ crystal structure were obtained from the RCSB-Protein Data Bank (PDB) [25] having PDB ID: 6LU7 [18]. The resolution and R-value of the selected M^pro^ were found to be 2.16 Å and 0.235, respectively. This protein consists of 306 amino acid residues with no mutation. To prepare the M^pro^ crystal structure, the “Protein Preparation Wizard” [26, 27] tool of Schrödinger suite was used. All water molecules and other heteroatoms were removed. Hydrogens were added, and missing atoms, side and backbone chains were corrected. Appropriate bond order and formal charges were adjusted. The protonation states of the protein were determined through PROPKA function of “Protein Preparation Wizard.” Using the OPLS3 forcefield, the M^pro^ protein was minimized to remove the steric clashes present in the protein structure. Thereafter, the prepared protein was considered for the grid generation using the “Receptor Grid Generation” panel of Glide (Grid-Based Ligand Docking with Energetics) [28] module of Schrödinger’s suite. For grid generation, the information of the catalytic active site and substrate binding site residues coordinates was used and thereby coordinates defined as −12.0, 18.0, 65.0 Å along *X*-, *Y*- and *Z*-axes, respectively. Grid box dimension was considered as 26 × 26 × 26 Å along *X*-, *Y*- and *Z*-axes, respectively. During grid generation, it was manually inspected and confirmed that all catalytic active site residues (such as His41 and Cys145) and substrate binding site residues were properly confined within the rectangular grid box.

### Virtual screening of chemical databases

Herein, to screen all three anti-viral specific chemical libraries, the employed DBVS protocol comprises three levels of molecular docking such as Glide-HTVS (high-throughput virtual screening), Glide-SP (standard precision) and Glide-XP (extra precision) and implemented under “Virtual Screening Workflow” (VSW) utility tool in the Maestro interface of Schrödinger’s suite [29]. Precisely, the VSW protocol is comprised of three consecutive docking methods/approaches where the outcome of one docking method becomes the input of the next level of the docking method and so on. In each docking approach, a user-defined certain percentage of docked molecules can be retained. The Glide uses the Emodel scoring function [28] which has much weightage to pick the “best” pose of a ligand. The main components of Emodel are Glide score and protein–ligand coulomb-vdW energy. The Glide score is an important function used to identify the active over inactive molecules. The QikProp module was checked to wipe out non-drug-likeness molecules. Molecules that remained after the final approach of docking such as Glide-XP were considered for binding energy calculation through Prime-MM-GBSA approach. Based on user-defined XP-score and binding free energy, top-ranked molecules were considered for further assessment.

### In silico pharmacokinetic and toxicity analyses

Molecules that remained after VSW approach were considered for pharmacokinetic assessment through the online SwissADME web server tool [30]. A number of parameters such as pharmacokinetic and drug-likeness were recorded. These parameters included physiochemical, lipophilicity, water-solubility, pharmacokinetic, drug-like properties, Lipinski’s rule of five (ROF) [31] and Veber’s rule [32] were calculated. The drug-likeness can be assessed using ROF which stated that to be a potential drug-like molecule, molecular weight, hydrophobicity, hydrogen bond (HB) acceptors and HB donors should not be more than 500 kDa, 5, 10 and 5, respectively. For being a promising drug-like molecule, Veber’s rule explained that total polar surface area (TPSA) and the number of rotatable bonds should not be higher than 140Å^2^ and 10, respectively. Two crucial pharmacokinetic parameters such as human intestinal absorption (HIA) and blood–brain barrier (BBB) can also be used to select drug-like molecules [33]. The percentage of absorption by the intestine can be assessed using the HIA parameter [33]. The penetrability competence of the molecule in the brain can be estimated using BBB parameter.

On the other hand, the pkCSM, a web server tool [34], was used to evaluate the toxicity of the selected molecules. This tool is widely used by research communities across the globe due to the mathematical formulation integrated in terms of graph-based signatures algorithm to generate predictive models of different pharmacokinetics and toxicity properties for any given molecule. A number of parameters related to the toxicity including AMES toxicity, maximum tolerated dose (human), hERG-I/hERG-II inhibitor, oral rat acute toxicity, oral rat chronic toxicity (LOAEL), hepatotoxicity, skin sensitization toxicity were generated after the SMILES formatted input of each compound. The above parameters are extremely essential to evaluate the toxicity for drug-like molecules, and the molecules having values in unacceptable range for further assessment can be removed.

### Molecular dynamics simulation

MD is an excellent computer simulation approach that is highly being explored in the field of drug discovery research to understand behavioral changes of the protein–ligand complex in the dynamic environment at the atomic level. It is also an essential tool to evaluate the intra- or interatomic interaction stability of the protein–ligand complex against user-defined specified time span. In the present study, four promising small molecules complex with M^pro^ protein were subjected to 100 ns classical MD simulation production run. The MD simulation execution was performed in the Desmond platform [35] integrated into the Schrödinger suite. Each protein–ligand complex system was confined within the orthorhombic box having the size of 10 × 10 × 10 Å. The TIP3P water model [36] was used to solvate the system. The appropriate number of counter ions was adjusted to neutralize the system. The salt concentration was maintained as 0.15 M. Followed by setting up the system builder, each system was minimized through the Steepest Descent followed by limited memory Broyden–Fletcher–Goldfarb–Shanno (LBFGS) algorithms with maximum iterations of 2000. The short-range coulombic interactions were evaluated using the cutoff radius of 9 Å. On the other hand, long-range coulombic interactions cutoff was considered through the smooth particle mesh Ewald method (PME) [37]. To equally distribute the ions and solvent around the protein–ligand complex, each system was equilibrated using the NPT (*N*—number of the particle, *P*—system pressure, *T*—temperature) ensemble with Nose–Hoover chain thermostat. The temperature was kept constant at 300 K throughout the simulation. The Langevin thermostat was used to regulate the temperature with a relaxation time of 1 ps span. Moreover, 2 ps relaxation time of barostat pressure was regulated by isotropic position scaling. A total of 1000 frames were saved for the simulation system for further analysis using “Simulation Interactions Diagram” and “Simulation Event Analysis” modules embedded in the Desmond program. On successful completion of the MD simulation, a number of parameters including RMSD of the protein backbone and ligand, root-mean-square fluctuation (RMSF) of individual amino acids and atoms of ligand, and, the radius of gyration (RoG) were calculated from each MD simulation trajectory.

The binding free energy of each proposed small molecules was estimated through MM-GBSA approach. This approach is widely used and trustworthy to calculate the binding affinity of the molecules towards the target biomacromolecules. The detailed methodology can be found in our previous publications [38, 39].

## Results and discussion

### Virtual screening of chemical databases

**Fig. 1:**
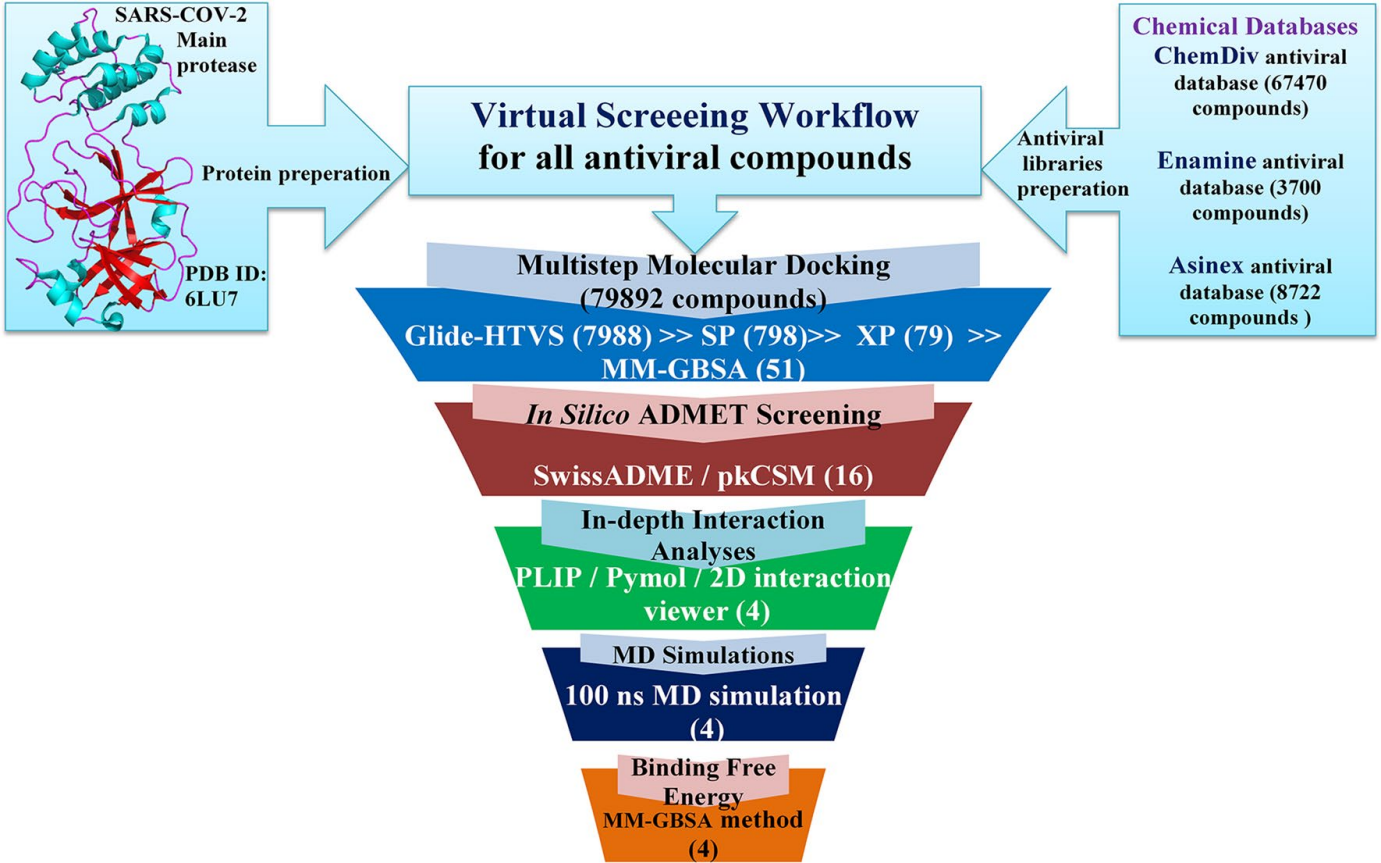
Schematic representation of virtual screening of chemical databases against M^pro^

The molecular docking has become an important and integrated protocol for SBVS of chemical databases to find out the promising molecules for a specific target. Due to its computationally cheaper and trustworthy nature and availability of extensive parameter selection features, it is widely used in the pharmaceutical research community. In the current study, considering the M^pro^ as the target molecule, initially three levels of molecular dockings, such as Glide-HTVS, Glide-SP and Glide-XP, were executed to screen the entire anti-viral chemical libraries of ChemDiv, Asinex and Enamine databases consisting of 79,892 compounds. A stepwise flow diagram of the presented work is given in Fig. 1.

**Fig. 2:**
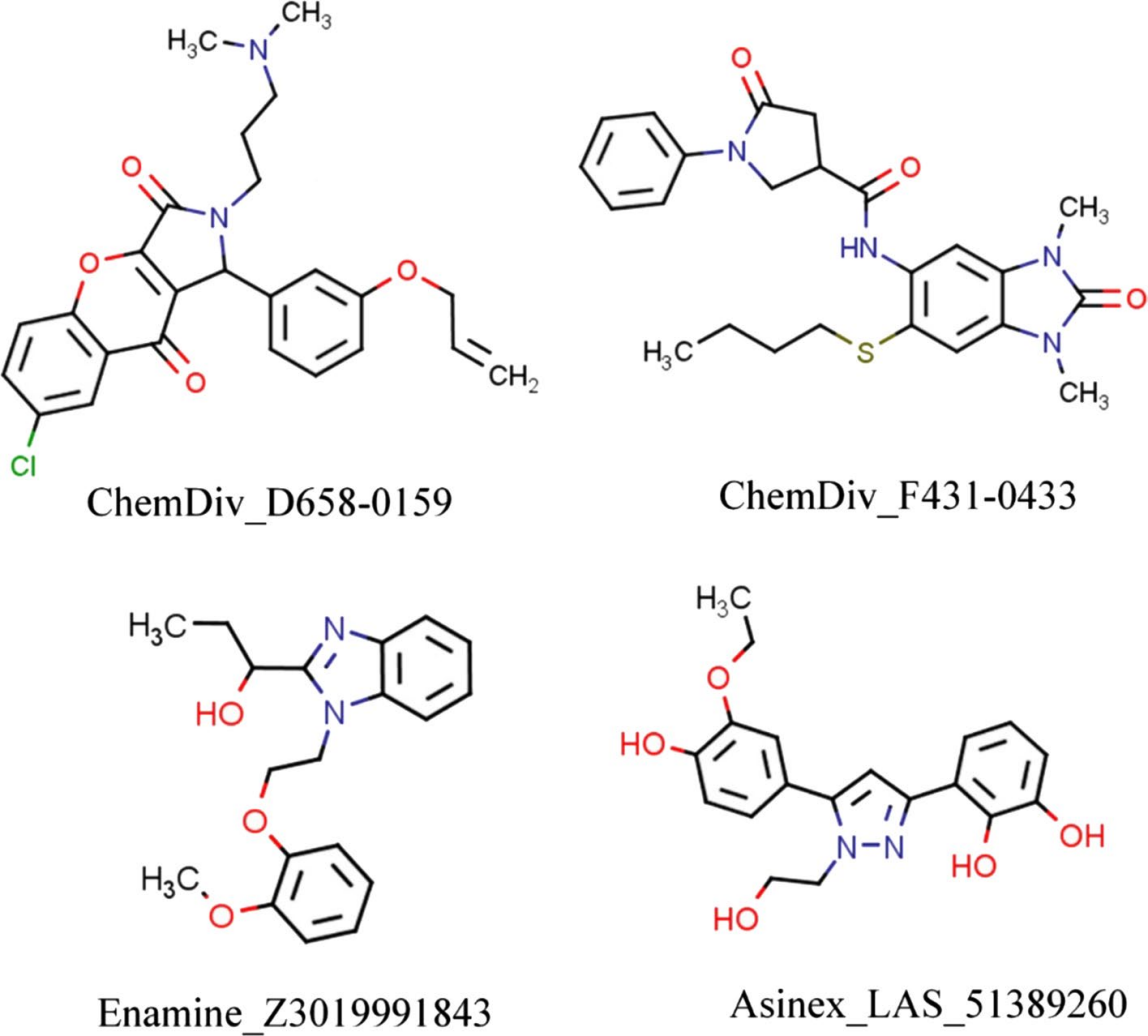
Two-dimensional (2D) representation of proposed M^pro^ inhibitors. Each molecule was found to consist of crucial pharmacophoric features

The VSW module is the successive filtering approach in which relatively inactive molecules are filtered out in each step of Glide-HTVS, Glide-SP and Glide-XP docking, sequentially. Herein, from each step of docking top 10% docked molecules (based on Glide dock score) were considered for the next level of docking assessment. Therefore, in the first step, altogether molecules of three databases (i.e., 79,892 compounds) were given as input in Glide-HTVS and a total of ~ 7988 molecules were retained after completion of the Glide-HTVS docking step. Thereafter, the above-retained number of molecules (~ 7988) were again used as input for the Glide-SP docking and the top 10% compounds (i.e., 798) docked compounds were obtained/retained. Finally, retained molecules in the previous step were further docked through Glide-XP and the top 10% molecules were considered for subsequent analysis. The XP-dock score of each retained molecule was recorded, and it was found to be in the range of −5.27 to −9.51 kcal/mol. A total of 79 molecules were found in the preceding step and were further subjected to binding free energy calculation through the Prime-MMGBSA approach. For further reduction of chemical space, a user-defined cut-off XP-score and Prime-MMGBSA-based binding free energy were considered as −8.00 and −45.00 kcal/mol, respectively. It was observed that a total of 28 molecules were failed to satisfy the above criteria. Therefore, the remaining 51 molecules were subjected to *in silico* pharmacokinetic and toxicity assessment through SwissADME [30] and pkCSM [34] web server tools. On detailed analysis, it was found that 16 molecules possess acceptable ADME and toxicity profiles. The binding interactions of each molecule retained in the previous step were explored in detail using the PLIP (Protein–Ligand Interaction Profiler)—a web-based server [40] widely used for characterization of interactions for any given protein–ligand complexes. Four molecules were found to have crucial binding interactions with catalytic or active site residues of M^pro^ protein by means of formation of several numbers of hydrogen bond and hydrophobic contacts, and salt bridge interaction, etc., and hence, considered to be promising M^pro^ inhibitors for SARS-CoV-2 inhibition. It is an important matter to mention that among all four proposed molecules, two are from ChemDiv database (ChemDiv_D658-0159 and ChemDiv_F431-0433) and one of each from Enamine (Enamine_Z3019991843) and Asinex (Asinex_LAS_51389260) databases. The 2D chemical representation of the final selected molecules is given in Fig. 2. On close inspection, it was found that all molecules consisting of a diverse type of functional groups or pharmacophoric features might be crucial to form binding interactions with catalytic amino residues of M^pro^.

### Binding interactions analysis

The Glide XP-dock score and Prime MM-GBSA-based binding free energies of the proposed molecules were found to be −8.59 and −61.67 kcal/mol; −8.96 and −62.85 kcal/mol; −8.44 and −58.86 kcal/mol; and, −8.49 and −61.06 kcal/mol for ChemDiv_D658-0159, ChemDiv_F431-0433, Enamine_Z3019991843 and Asinex_LAS_51389260, respectively. A number of biologically important amino acid residues of M^pro^ were found to be interacted with all the proposed molecules. The list of ligand-interacting amino acids along with XP-score and Prime-MMGBSA binding free energy is given in Table 1. It is important to note that XP-score was varied from −8.44 to −8.96 kcal/mol, and binding free energy was found in the range of −58.86 to −62.85 kcal/mol. The above-mentioned obtained energy scores clearly explained that all molecules possess somewhat energetically similar binding affinities towards the SARS-CoV-2 M^pro^ protein.

**Table 1:** XP-score, Prime-MMGBSA-based binding free energy and ligand-interacting amino acids

Compounds	Glide XP/MM-GBSA score (Kcal/mol)	Interacting residues in H-bond interaction	Other type of molecular interactions
ChemDiv_D658-0159	−8.59/−61.67	Asn142, Gly143, Glu166	Thr25, Leu27, Asn142, Met165, Gln189 (Hydrophobic) Glu166 (Salt bridge)
ChemDiv_F431-0433	−8.96/−62.85	Gly143, Glu166, Thr190	Thr25, His41, Met165, Gln189 (Hydrophobic)
Enamine_Z3019991843	−8.44/−58.86	Asn142, Gly143, Gln189	Thr25, His41, Met165, Glu166, Asp187 (Hydrophobic)
Asinex_LAS_51389260	−8.49/−61.06	Thr26, His41, Tyr54, Glu166	Met165, Glu166, Gln189 (Hydrophobic)

**Fig. 3:**
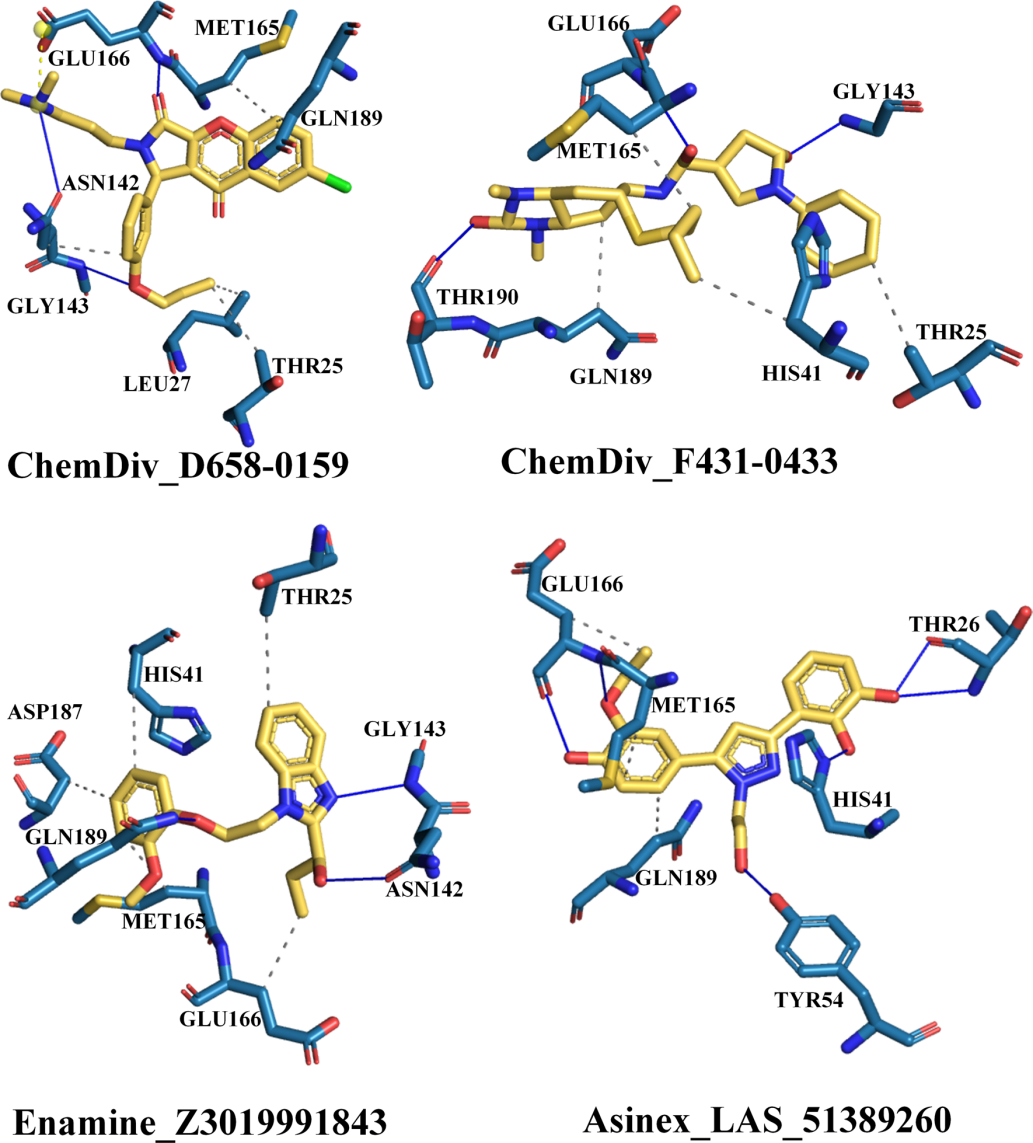
Binding interaction profile of M^pro^ protein with selected inhibitors/modulators. Solid lines (in blue color) are represented as hydrogen bonds and dotted lines are as hydrophobic interactions (in gray color) and yellow dotted line as salt bridge interaction

The intermolecular binding interactions of each proposed molecule were assessed, and interactions profiles are given in Fig. 3. Particularly, residues Asn142 and Gly143 were found to form hydrogen bond interactions with both compounds ChemDiv_D658-0159 and Enamine_Z3019991843. In addition, ChemDiv_F431-0433 was found to interact with residue Gly143 through a hydrogen bond interaction. Another acidic amino acid Glu166 was critically formed hydrogen bond interaction and hydrophobic contacts with both the compounds ChemDiv_D658-0159 and Asinex_LAS_51389260. Moreover, Glu166 was also observed to establish hydrogen bond and hydrophobic interactions with ChemDiv_F431-0433 and Enamine_Z3019991843, respectively. Only Enamine_Z3019991843 created a hydrogen bond interaction with residue Gln189, while same amino acid residue establishes hydrophobic interactions with all other three proposed compounds. Polar amino acid residue, Thr190 interacted with ChemDiv_F431-0433 through a hydrogen bond interaction. Asinex_LAS_51389260 possessed a slightly different kind of binding interaction profile in comparison with the other three proposed compounds. Three other amino acids including one catalytic residue of M^pro^ such as Thr26, His41 and Tyr54 successfully interacted with Asinex_LAS_51389260 through hydrogen bond interactions. Docking analyses revealed that amino acid residue Thr25 was found to be a common residue for establishing hydrophobic contacts with various atoms or functional groups of compounds ChemDiv_D658-0159, ChemDiv_F431-0433 and Enamine_Z3019991843. The basic and active site residue His41 of M^pro^ protein was critically formed hydrophobic interactions with compounds ChemDiv_F431-0433 and Enamine_Z3019991843. Another amino acid residue Met165 was also observed to form hydrophobic contacts with all four proposed inhibitors. Beyond the above-mentioned residues association in interaction participation with SARS-CoV-2 M^pro^ protein, residue Asp187 was found to form a hydrophobic contact with compound Enamine_Z3019991843. All the observed binding interactions certainly demonstrated the efficiency and presence of numbers of hydrogen bond acceptors and donors group/atoms in the proposed M^pro^ inhibitors. Majorly, all critical binding interactions found between the proposed inhibitors/modulators and catalytic amino acid residues of M^pro^ might play a crucial role by means of holding all the ligands inside the receptor cavity tightly and to exhibit some level of energetically profound binding kinetics underlying the protein–ligand binding mechanism and hence can display desirable therapeutic or pharmacologic effect.

**Fig. 4:**
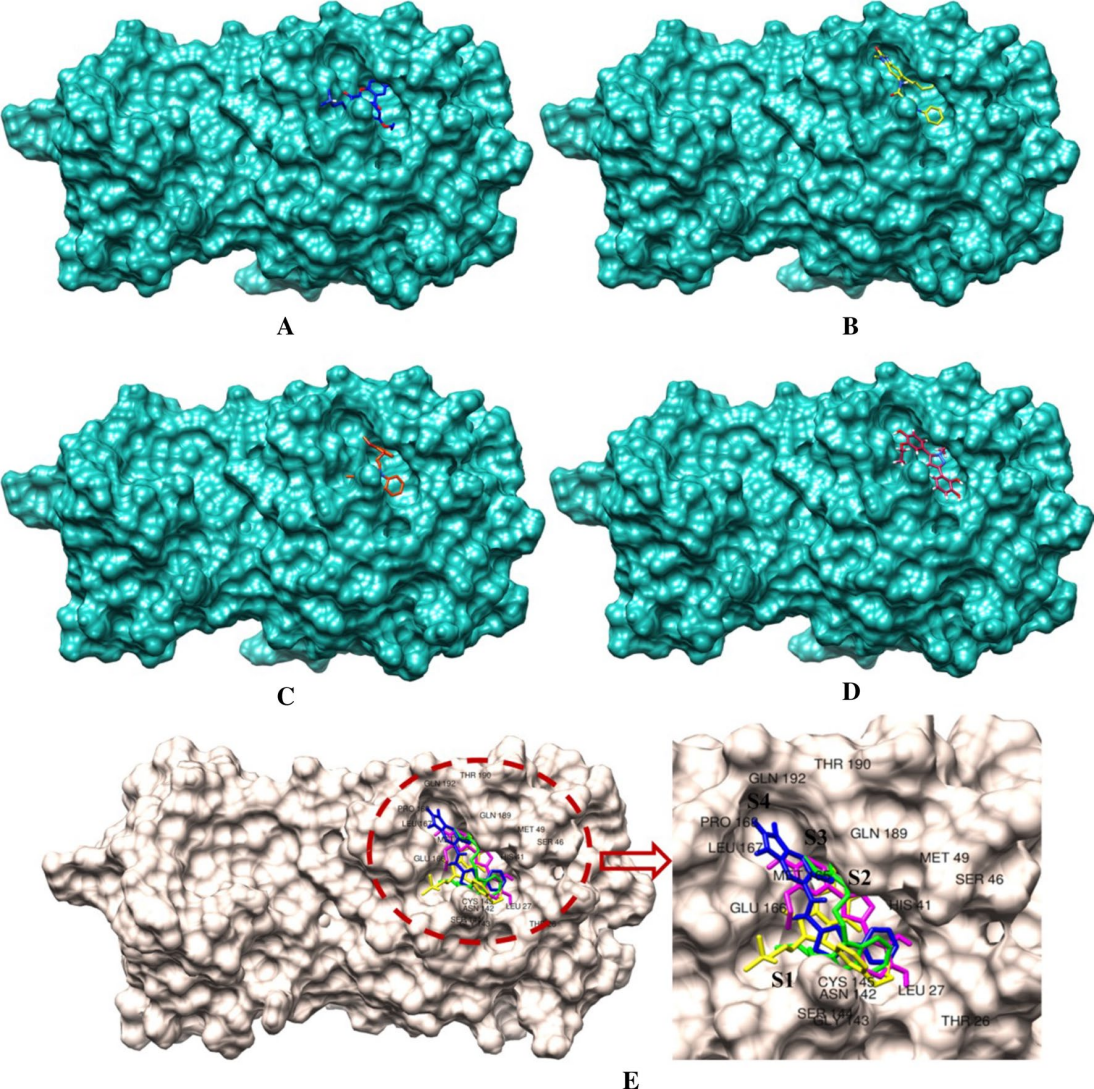
Binding mode of four proposed M^pro^ inhibitors at the active and substrate binding sites displayed in surface view representation. Four substrate binding sites (S1-S4) are marked. **a** ChemDiv_D658-0159 in yellow, **b** ChemDiv_F431-0433 in blue, **c** Enamine_Z3019991843 in green, **d** Asinex_LAS_51389260 in magenta colors, **e**. Binding mode of all selected molecules in different subdivision of substrate binding sites/pockets of SARS-COV-2 M^pro^ protein

For better understanding, the binding mode of each molecule, the detailed geometric orientation of all molecules in the active site cavity or its close vicinity of M^pro^ protein was generated as 3D surface view presentation and is depicted in Figs. 4a–d. It can be seen that each of the selected molecules was perfectly fitted inside the receptor cavity of M^pro^ protein. From the intermolecular interaction profiles and binding mode analyses of each molecule, it can be postulated that all selected molecules probably accorded its active state conformation and hence possibly can exhibit essential inhibition or modulation effect to the M^pro^ protein.

The binding site subdivision of M^pro^ was reported by Zhang and associates [13] with four different regions termed as S1, S2, S3 and S4. The binding mode inside the different binding site subdivision of the selected molecules is given in Fig. 4e. The S1 site was created with the amino acid residues Phe140, Asn142, Ser144, Cys145, His163, His172, and Glu166, and the backbone of Leu141, Gly143, His164, and Met165. The side chains of residues His41, Val42, Asn119, Thr25, Cys145, Gly143, and the backbone of residue Thr26 were involved in S2 sub-binding site. Subsite S3 was created by the side chains of Tyr54, Asp187, Met49, and His41 as well as the backbone of Arg188. Subsite S4 was involved with the side chains of residues Met165, Leu167, Pro168, Ala191, and Gln192, and the backbones of residues Glu166, Arg188, and Thr190 [41]. It is interesting to note that all proposed molecules were involved in binding interactions with at-least one amino acid residue from each subsite. The above observation was explained that proposed molecules perfectly occupied the entire binding site of the M^pro^ which increases the probability for the formation of biologically relevant interactions and hence inhibition or modulation of protein activity can be achieved.

Recently, Mazzini et al. [42] have explored the screening of an in-house library of natural and nature-inspired products to identify promising M^pro^ inhibitors. The said study reported nine potential molecules through structure-based virtual screening. The binding profile reported in the mentioned work was found to be almost similar in terms of binding interaction outcomes found in the present study. Particularly, few crucial amino acids, such as His41, Leu27, Asn142, Gly143, Cys145, Met165, Glu166, Gln189, and Thr190, were revealed as critical residues for the creation of intermolecular interaction with the proposed molecules. The majority of the above-mentioned amino acid residues M^pro^ were also found to be common residues that interacted with all the proposed compounds, obtained in the current study. In another study, Gahlawat et al. [43] performed a comparative study to check the insight of the mutations at the active site followed by the screening of natural and drug databases to propose potential M^pro^ inhibitors. In that study, authors explored binding interactions between proposed molecules and M^pro^. Interestingly, all the catalytic amino acid residues found to interact with their reported molecules are also found to be common residues in interactions creation obtained in our current study outcomes. Another study by Kanhed et al. [44] was performed to screen the drug library and Asinex database against the M^pro^ protein. The XP-docking score of the proposed molecules was found to be relatively similar as the present study findings. In that reported molecular docking analyses, the authors mentioned few important catalytic amino acid residues that are Thr25, His41, Asn142, Gly143, Met165, Glu166, Gln189 and Thr190, etc., crucial for the formation of binding interactions with the M^pro^ and postulated for biological inhibition of that proteolytic enzyme. Interestingly, the majority of the substrate-binding site residues were found to interact with the reported molecules except for non-involvement of one catalytic site residue Cys145. In such similar fashion, our docking study outcomes also demonstrated somewhat alike binding interactions maps for all proposed molecules where the absence of any intermolecular interaction with the residue Cys145 was observed. The appearance of such observation might be due to protein flexibility issues. Because the XP-docking was conducted following the rigid receptor docking protocol where flexibility to the side or backbone chain to any of amino acids was not provided. In some other study, where the drug re-purpose approach through a combined ligand- and structure-based screening was presented by Ferraz et al. [17] and finally, two oral and one buccal drug were identified as effective molecules against the M^pro^. The above-mentioned study also reported similar binding interaction profiles as obtained in the docking analyses of the current study.

### Pharmacokinetic and toxicity assessment

The SwissADME online tool was used to calculate the pharmacokinetic and drug-likeness characteristics, and these are given in Table 2. All selected molecules were found to possess highly absorbable to the GI and soluble in nature. Not a single molecule was found to violate ROF, Veber’s and Ghose's rules. The molecular weight (MW) of the molecules was found to be in the range of 325 to 453 g/mol that fairly suggested good absorption. The total polar surface area (TPSA) less than 140 Å^2^ of any molecule suggested good intestinal permeability. All four selected molecules were found to have TPSA less than 108 Å^2^ that indicated good permeability in the intestine. The number of rotatable bonds was found to be 8, 8, 7 and 6 for ChemDiv_D658-0159, ChemDiv_F431-0433, Enamine_Z3019991843 and Asinex_LAS_51389260, respectively, that indicated the reasonable rigidity and flexibility of the molecules. The synthetic accessibility of the molecules was observed to be in the range of 3 to 4.30. The above observation of synthetic accessibility undoubtedly suggested that molecules can easily be synthesized.

**Table 2:** Predicted ADMET profiles of selected four SARS-CoV-2 M^pro^ inhibitors/modulators compounds

Parameters	ChemDiv_D658-0159	ChemDiv_F431-0433	Enamine_Z3019991843	Asinex_LAS_51389260
^1^ MW (g/mol)	452.93	452.57	326.39	356.37
^2^NHA	32	32	24	26
^3^NAHA	16	15	15	17
^4^NRB	8	8	7	6
^5^TPSA (Å^2^)	62.99	101.64	56.51	107.97
^6^LogS	−5.09	−4.1	−3.81	−3.45
^7^SC	Highly	Highly	Moderately	Highly
	Soluble	Soluble	Soluble	Soluble
^8^GI	High	High	High	High
^9^BBB	No	No	No	No
^10^vROF	0	0	0	0
^11^vGhose	0	0	0	0
^12^vVeber	0	0	0	0
^13^BS	0.55	0.55	0.55	0.55
^14^SA	3.25	3.82	3.30	3.03
iLOGp	4.18	3.78	3.09	3.14

^1^Molecular weight; ^2^No.of heavy atoms; ^3^No. of aromatic heavy atoms; ^4^No. of rotatable bonds; ^5^Topological polar surface area; ^6^Solubility; ^7^Solubility class; ^8^Gastrointestinal absorption; ^9^Blood–brain barrier penetration; ^10^Violation of Lipinski’s rule of five; ^11^Violation of Ghose's rule; ^12^Violation of Veber’s rule; ^13^Bioavailability score; ^14^Synthetic accessibility

Further, the toxicity assessment was carried out for the selected M^pro^ inhibitors through pkCSM web server tool. Value of all the generated parameters related to the toxicity is listed in Table 3. The AMES toxicity parameter explained that all molecules were non-mutagenic in nature. The low value of maximum tolerated toxic dose clearly indicated the non-toxicity behavior of the molecules. Not a single molecule was found to have ventricular arrhythmia characteristics which were substantiated by hERG I/hERG II inhibitor parameter. Moreover, a safety concern for drug-induced liver injury was measured through the hepatotoxic indication for all compounds, which bring out as negative that clearly indicated no disruption of normal function of the liver upon administration. The skin sensitivity of the molecules was found to be negative. The oral rat acute toxicity (LD_50_) was found to be 2.69, 2.68, 2.24 and 2.42 for ChemDiv_D658-0159, ChemDiv_F431-0433, Enamine_Z3019991843 and Asinex_LAS_51389260, respectively. The oral rat chronic toxicity (LOAEL) of the molecules was found in the range of 0.9 to 1.60. Both LD_50_ and LOAEL were found within the recommended value. Therefore, the above discussion and values of ADME and toxicity were revealed that all the selected inhibitors showed potential lead-like features and can be used for further estimation for SARS-CoV-2 M^pro^ modulation and biological activity.

**Table 3:** Predicted toxicity of selected Mpro inhibitors

Toxicity properties	ChemDiv_D658-0159	ChemDiv_F431-0433	Enamine_Z3019991843	Asinex_LAS_51389260
AMES toxicity	No	No	No	No
Max. tolerated dose (human)	0.20	0.46	0.64	0.61
hERG I/hERG II inhibitor	No/No	No/No	No/No	No/No
Oral rat acute toxicity (LD_50_)	2.69	2.68	2.24	2.42
Oral rat chronic toxicity (LOAEL)	0.95	1.29	1.46	1.60
Hepatotoxicity	No	No	No	No
Skin sensitization	No	No	No	No

### Molecular dynamics simulation analyses

The dynamic behavioral characteristics of the proposed molecule-SARS-CoV-2 M^pro^ protein complexes were explored through 100 ns all-atoms MD simulations study. Particularly, to assess the protein–ligand complex stability, a number of parameters including protein backbone RMSD, ligand RMSD, RMSF of each amino acid residue, atomic RMSF of each ligand and protein backbone RoG were estimated from the MD simulation trajectories. Maximum, minimum and average values of all above parameters were calculated and are listed in Table 4.

**Table 4:** Maximum, minimum and average RMSD, RMSF and RoG are estimated from the MD simulation trajectories

			ChemDiv_D658-0159	ChemDiv_F431-0433	Enamine_Z3019991843	Asinex_LAS_51389260
RMSD (Å)	M^pro^ backbone	Minimum	0.86	1.07	1.06	0.89
		Maximum	5.87	2.94	2.51	2.66
		Average	2.38	2.21	1.78	1.71
	Ligand	Minimum	0.62	0.50	0.30	0.74
		Maximum	1.38	2.22	2.32	2.87
		Average	0.95	1.19	1.29	2.16
RMSF (Å)	Amino acids	Minimum	0.43	0.36	0.37	0.38
		Maximum	3.70	2.96	2.66	2.89
		Average	1.67	0.88	0.88	0.97
	Ligand	Minimum	0.19	0.33	0.30	0.49
		Maximum	1.52	1.81	2.22	1.94
		Average	0.46	0.88	0.95	1.07
Protein RoG (Å)		Minimum	4.25	4.41	3.38	3.62
		Maximum	4.74	5.28	4.17	4.48
		Average	4.54	4.90	3.85	4.14

#### Root-mean-square deviation analyses

**Fig. 5:**
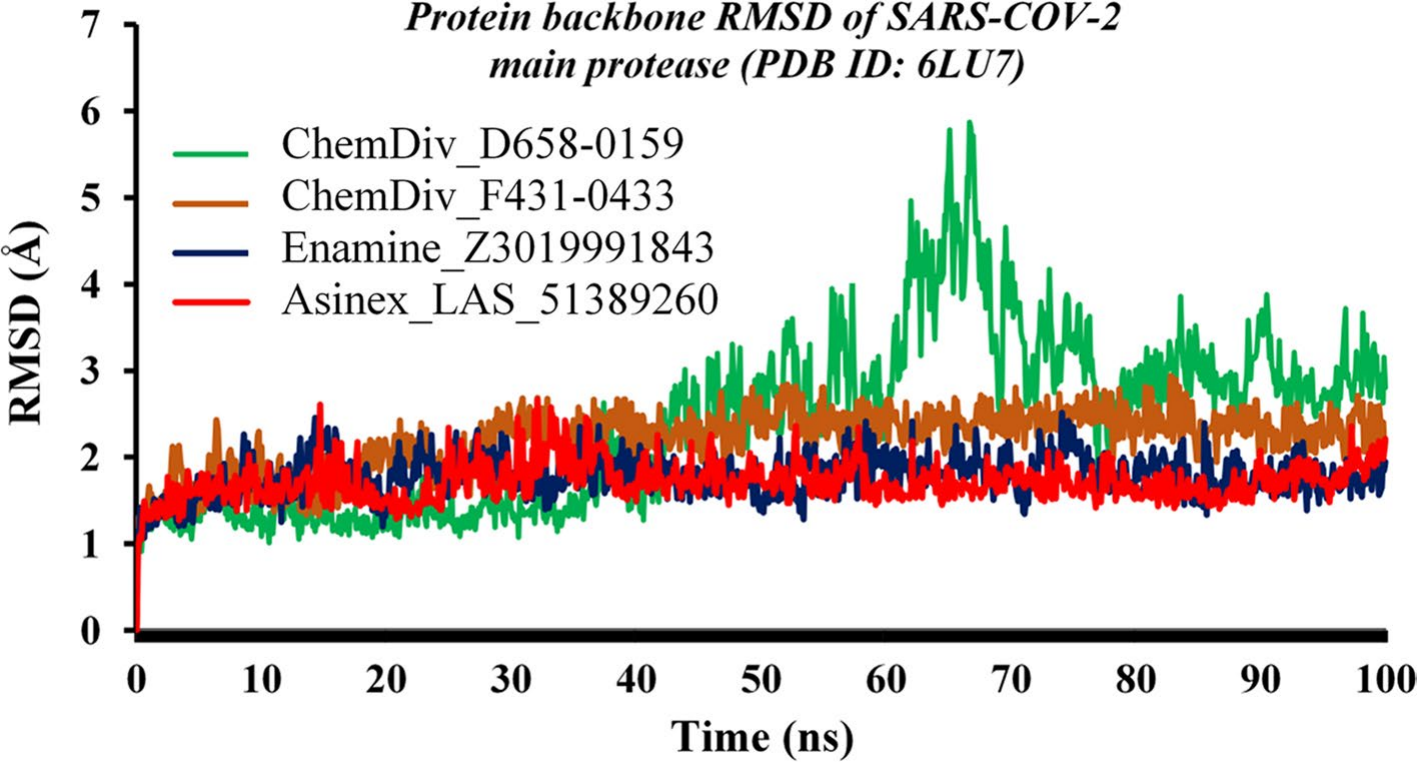
M^pro^ backbone RMSD bound with ChemDiv_D658-0159, ChemDiv_F431-0433, Enamine_Z3019991843 and Asinex_LAS_51389260. The consistent protein backbone RMSDs exhibited equilibration of each protein–ligand complex system throughout the simulation

The deviation of the SARS-CoV-2 M^pro^ protein backbone bound with all proposed inhibitors was assessed through the protein backbone RMSDs. M^pro^ backbone RMSD values of each frame obtained through MD simulation production bound with all four molecules were plotted against the time scale, and it is given in Fig. 5. The consistent RMSD values with lower deviation in the M^pro^ backbone bound with three proposed inhibitors, viz*.* ChemDiv_F431-0433, Enamine_Z3019991843 and Asinex_LAS_51389260, throughout the simulation run clearly indicated the interactions stability of the M^pro^ protein–ligand complexes. On the other hand, M^pro^ backbone bound with compound ChemDiv_D658-0159 was found to deviate for a short time span, ~ at 60–70 ns and immediately thereafter RMSD values were found to be progressing towards their equilibrium state. From Table 3, the average RMSD of M^pro^ backbone bound with ChemDiv_D658-0159, ChemDiv_F431-0433, Enamine_Z3019991843 and Asinex_LAS_51389260 was found to be 2.38, 2.21, 1.78 and 1.71 Å, respectively.

**Fig. 6:**
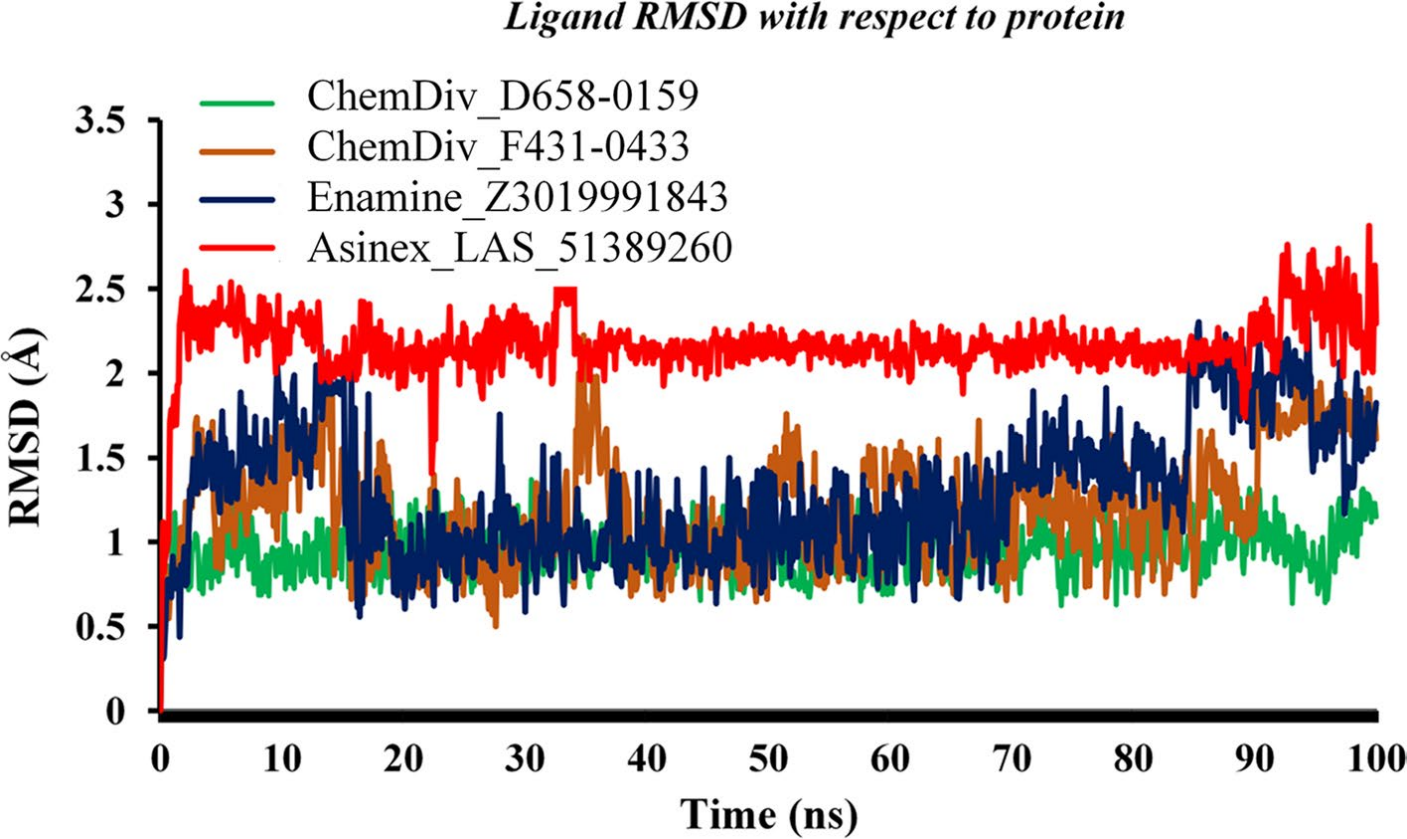
RMSD values of each proposed SARS-CoV-2 M^pro^ inhibitor/modulator vs. time of the simulation

The RMSDs of all proposed ligands were also assessed through the entire MD simulation trajectories. For all four proposed molecules, the RMSD values were plotted against the time of simulation and it is given in Fig. 6. Initially, both ChemDiv_F431-0433 and Enamine_Z3019991843 have changed the conformational orientation and deviated up to about 2 Å. The same trend persisted till ~ 18 ns for both compounds and afterward regained conformational integrity as the initial or starting point. Interestingly, at ~ 85 ns, RMSD of both molecules deviated up to 2.5 Å. Such observations might be due to the opening and closing of the active site that gives sufficient space to the small molecule to achieve new orientations. ChemDiv_D658-0159 was found to retain an almost similar conformational state throughout the simulation run. The RMSD of Asinex_LAS_51389260 deviated at the very beginning of the simulation, and thereafter, it achieved equilibration and maintained its consistency till the simulation end. Overall, the above-mentioned low RMSD values for both the protein backbone and ligands undoubtedly suggested protein–ligand conformational stability in the dynamic state for all four compounds bound with M^pro^ protein.

#### Root-mean-square fluctuation analyses

**Fig. 7:**
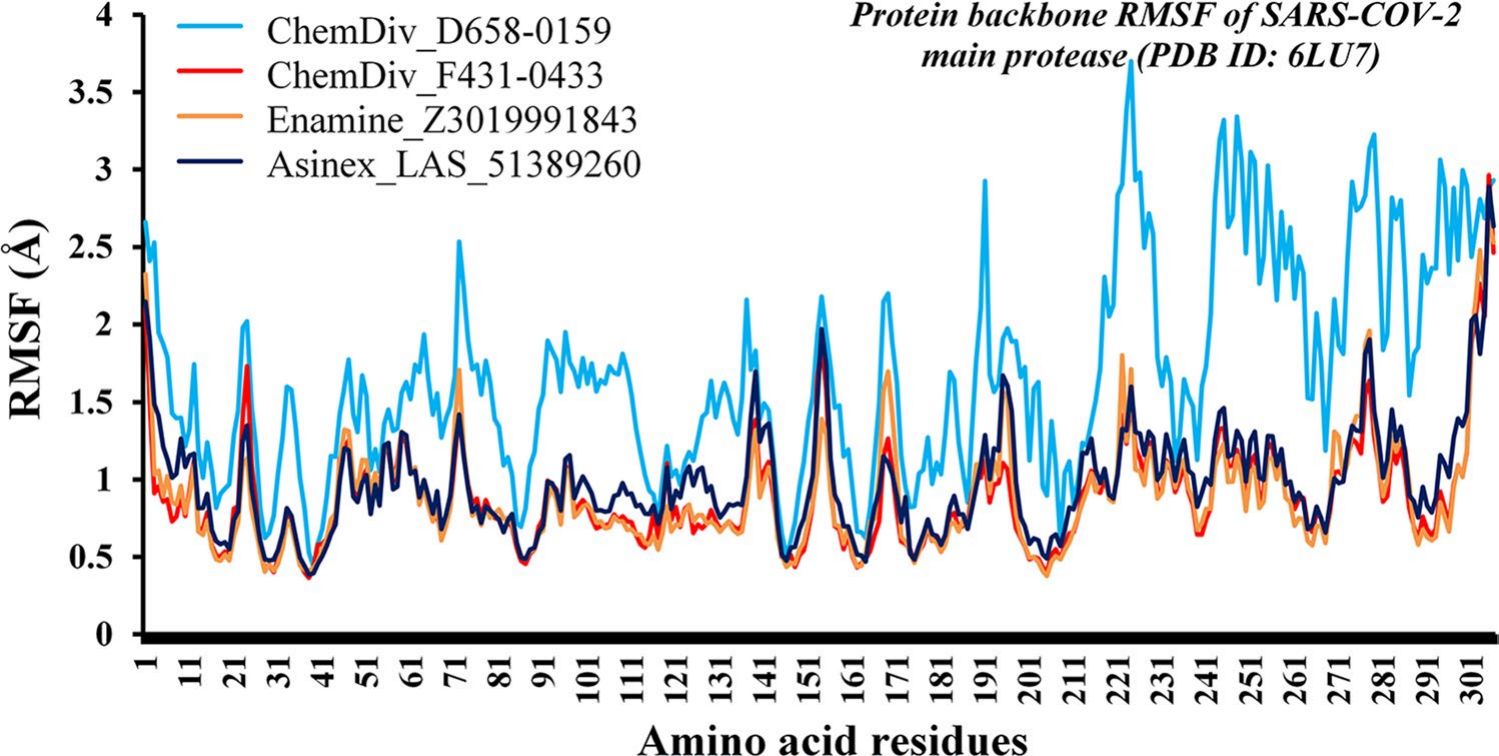
RMSF values of each amino acid residues of M^pro^ protein calculated from MD simulation trajectories

The structural integrity and amino acid residual mobility can be explained through the RMSF of each amino acid residue during the simulation. From the MD simulation trajectories, the RMSF of SARS-CoV-2 M^pro^ protein bound with ChemDiv_D658-0159, ChemDiv_F431-0433, Enamine_Z3019991843 and Asinex_LAS_51389260 was estimated and it is given in Fig. 7. The RMSF of the terminal amino acids was found to be higher as expected. A similar pattern of change in RMSF values of M^pro^ amino acid residues bound with compounds ChemDiv_F431-0433, Enamine_Z3019991843 and Asinex_LAS_51389260 was found. It is also important to note that RMSF of ligand-binding amino residues (Table 1) of the above complexes was found to be consistent and low in comparison with M^pro^ bound with ChemDiv_D658-0159. Such observation might be due to the existence or formation of less number of intermolecular binding interactions during MD simulations between ChemDiv_D658-0159 and key amino acid residues of SARS-CoV-2 M^pro^ protein in comparison with other three compounds. Although the RMSF values of M^pro^ protein bound with ChemDiv_D658-0159 were comprehended to fluctuate relatively higher scale (reached up-to ~ 3.70 Å) in contrast to three others compounds, but no abnormal deviations were observed in the RMSF values. It can be hypothesized that due to weak interactions between catalytic amino residues of M^pro^ and ChemDiv_D658-0159 such higher RMSF was observed. Average, maximum and minimum RMSF values of M^pro^ protein bound with ChemDiv_D658-0159, ChemDiv_F431-0433, Enamine_Z3019991843 and Asinex_LAS_51389260 are given in Table 3. The RMSF of all complexes was varied in between 0.36 and 3.70 Å. The difference between the maximum and average RMSF can give an idea about the extension in the fluctuation of the amino acid residues bound with proposed M^pro^ inhibitors/modulators. The difference between the maximum and average RMSF was found to be 2.03, 2.08, 1.78 and 1.92 Å for M^pro^ bound with ChemDiv_D658-0159, ChemDiv_F431-0433, Enamine_Z3019991843 and Asinex_LAS_51389260, respectively. The above-mentioned low deviations in RMSFs values undoubtedly explained that M^pro^ amino acids did not fluctuate much during the MD simulation.

**Fig. 8:**
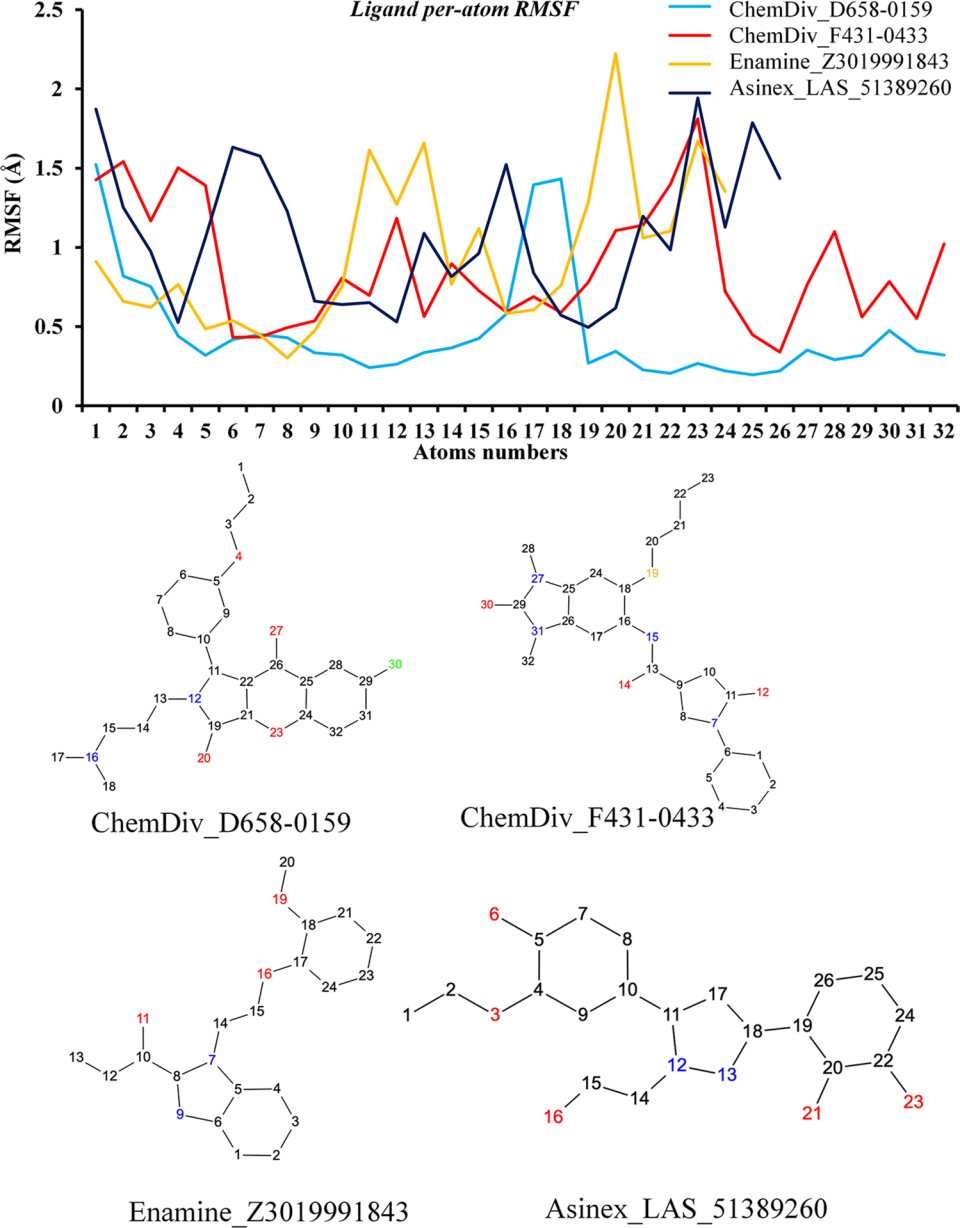
Atomic RMSFs of proposed compounds ChemDiv_D658-0159, ChemDiv_F431-0433, Enamine_Z3019991843 and Asinex_LAS_51389260 during MD simulation

Moreover, the atomic fluctuation of each ligand was also calculated and it is given in Fig. 8. The range of heavy atoms was found to be 1 to a maximum of 32. A distinct profile of atomic RMSF values was observed due to different structural moieties that hold all the proposed small molecules and also due to their different binding interactions profile in the dynamic state. It was observed that atoms of small molecules involved in binding interactions exhibited lower RMSF in comparison with the rest of the atoms which did not participate in interaction formation. Average RMSF values of compounds ChemDiv_D658-0159, ChemDiv_F431-0433, Enamine_Z3019991843 and Asinex_LAS_51389260 were found to be 0.46, 0.88, 0.95 and 1.08 Å, respectively. Such low atomic RMSF of the proposed molecules can certainly suggest conformational consistency and integrity during the MD simulation.

#### Radius of gyration (RoG) analyses

**Fig. 9:**
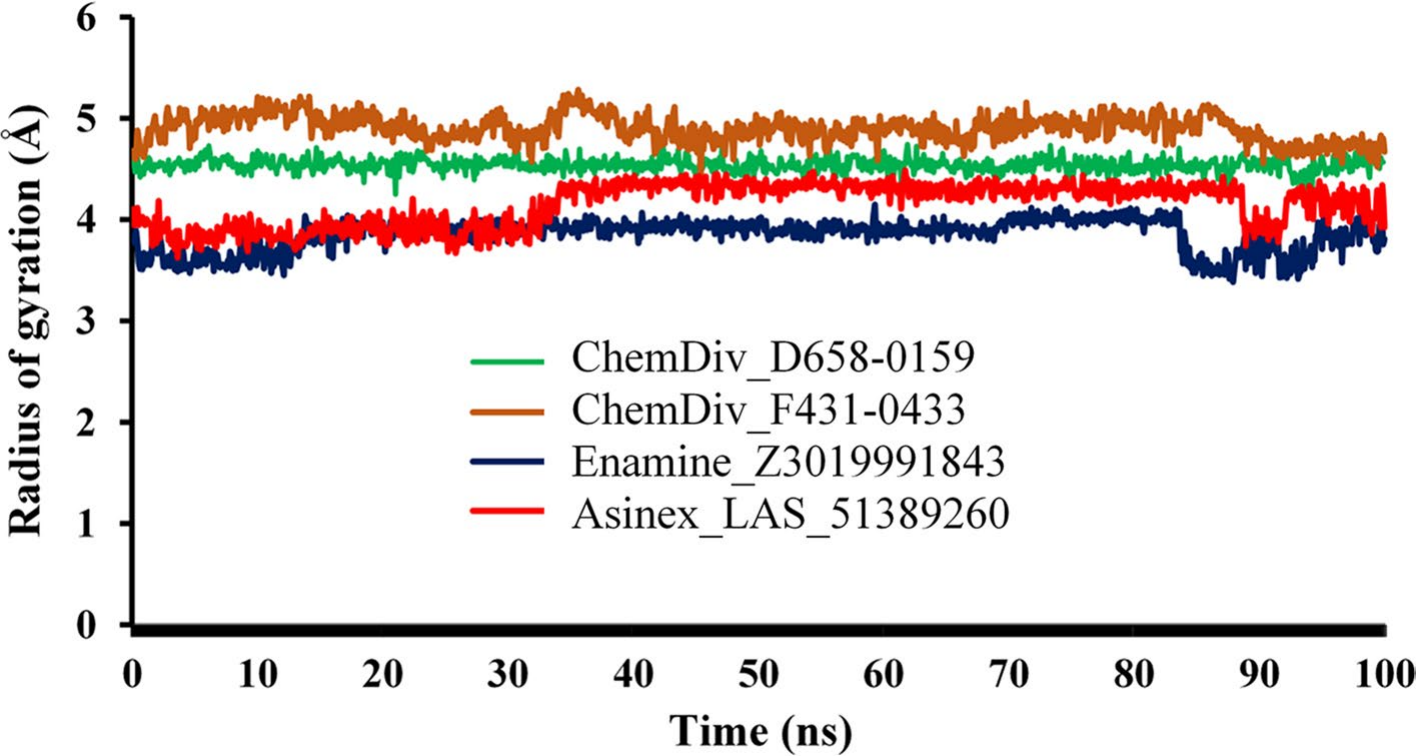
The RoG of Mpro bound with ChemDiv_D658-0159, ChemDiv_F431-0433, Enamine_Z3019991843 and Asinex_LAS_51389260

The compactness and rigidity of the M^pro^ protein bound with all proposed small molecules were explored through RoG, calculated from the MD simulation trajectories. The RoG of each frame against the time of the simulation is given in Fig. 9. Interestingly, very consistent RoG values were obtained in MD simulation analyses which certainly explains the stably folding nature of the protein during the entire MD simulation span. The RoG of M^pro^ bound with all four small molecules was seen very small magnitude of deviations throughout the simulation. The difference between the maximum and minimum RoG values of M^pro^ protein was found to be 0.49, 0.87, 0.79 and 0.86 Å, bound with proposed compounds ChemDiv_D658-0159, ChemDiv_F431-0433, Enamine_Z3019991843 and Asinex_LAS_51389260, respectively. The obtained RoG data can certainly explained the rigidity and compactness of M^pro^ protein structure during the MD simulation for all compounds bound state.

Earlier, Stoddard et al. [41] executed a research study employing drug repurposing approach to identify a few promising molecules for M^pro^ inhibition. The authors also performed MD simulation study of identified molecules bound with M^pro^ protein for 100 ns time span. The said study reported the RMSDs of M^pro^ backbone within the range of 0 to 4 Å. Further, the authors also calculated the RMSD of ligands and maximum value found within 4 Å. The authors concluded that MD simulation data were favored in the stability of protein–ligand complexes in the dynamic environment. As stated earlier, Kanhed et al. [44] also performed a screening of M^pro^ inhibitors and executed a relatively short (i.e., 10 ns) MD simulation study. RMSD of the protein backbone and ligand was noticed within 2 and 5 Å, respectively. The RMSF of M^pro^ amino acids was to be fluctuated around 1 Å. Kapusta et al. [45] explored the virtual screening of MolPort database and proposed about fifteen promising M^pro^ modulators. However, they have reported higher RMSD score (~ 6 to 10 Å) for M^pro^ protein backbone bound with few molecules. The available above-mentioned data in the literature successfully corroborated with the outcomes of the present study. Taken together, on the basis of RMSD of protein backbone and ligands, RMSF of amino acids and ligands and RoG values observed in the present study findings certainly can explain the protein–ligand complexes stability in dynamic condition.

### Protein–ligand contacts analyses during MD simulation

**Fig. 10:**
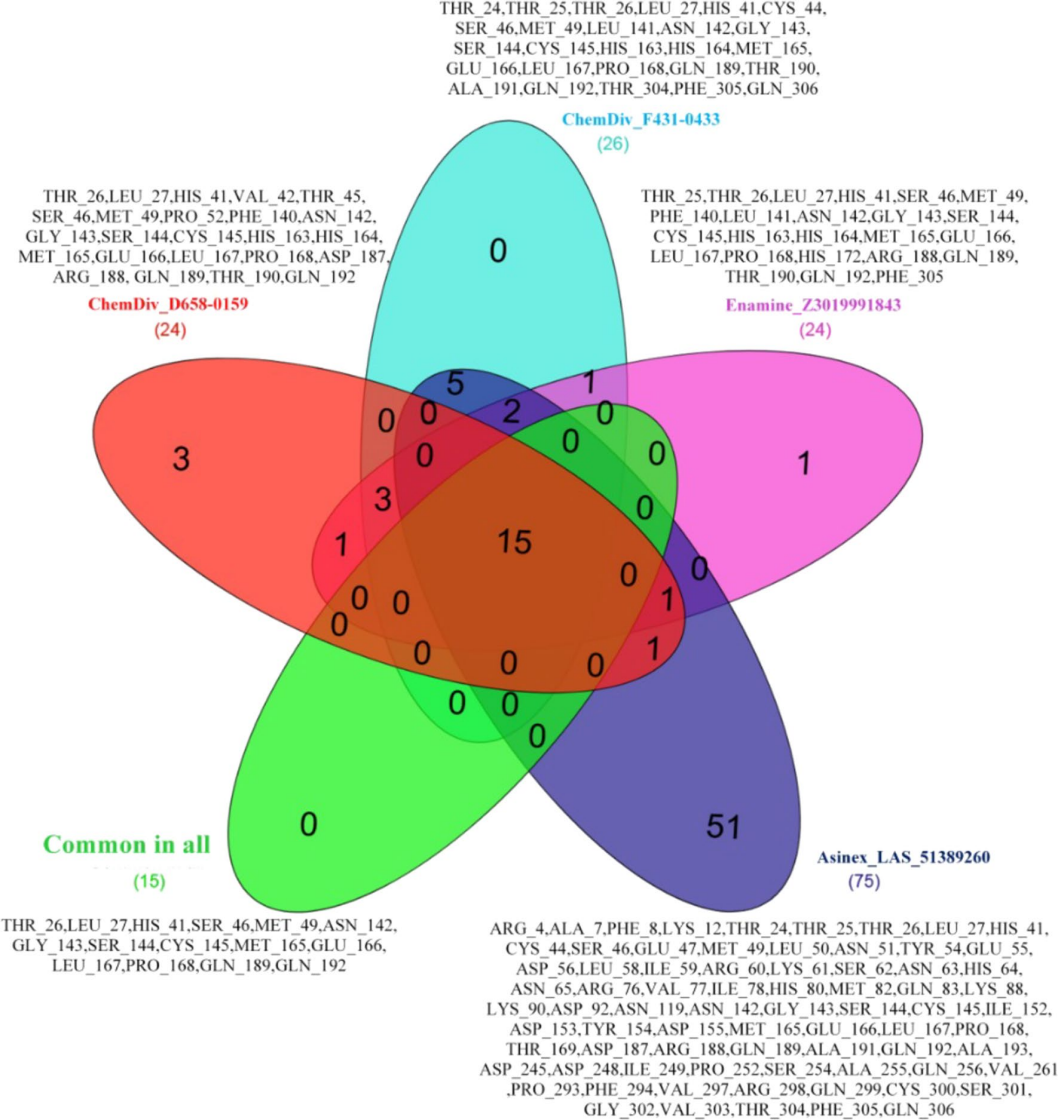
Venn diagram plot showing the ligand contacts with different residues of M^pro^ protein monitored throughout the simulation run

Moreover, the protein–ligand contacts/interactions were explored during MD simulation studies for all the complexes. Detailed analyses of protein–ligand contacts revealed that several ligand-M^pro^ protein atom pairs were created numbers of hydrogen, hydrophobic, ionic and water bridge interactions during MD simulation span and also maintained the same interactions profile for the certain time period. Particularly, several atoms of compounds ChemDiv_D658-0159, ChemDiv_F431-0433, Enamine_Z3019991843 and Asinex_LAS_51389260 were found to make contacts with at-least 24, 26, 24 and 75 different amino acid residues of M^pro^ protein, respectively, during MD simulation (Fig. 10).

From Fig. 10, it was interesting to note that at least 15 different amino acid residues (namely Thr26, Leu27, His41, Ser46, Met49, Asn142, Gly143, Ser144, Cys145, Met165, Glu166, Leu167, Pro168, Gln189 and Gln192) of M^pro^ were found to be as common residues to interact with all identified compounds in a dynamic state. Such important protein–ligand contacts monitored throughout the simulation undoubtedly suggested that all identified compounds specifically make multiple contacts at the substrate-binding site of M^pro^ protein and interactions with those residues are very much essential for inhibition or modulation of M^pro^ protein activity. Another important aspect was also noted from the protein–ligand contacts during simulations in terms of the appearance of new amino acid residues involvement in intermolecular interactions which did not appear in any docking based interaction analyses. Specifically, in docking-based analyses, the catalytic residue Cys145 was not found to form any types of interactions; however, MD simulation study confirmed the true presence and interaction frequencies of this important catalytic residue with all compounds. Such newly formed interactions explored from protein–ligand contacts might be observed due to governed flexibility and induce fit mechanism to both the ligands and protein as well, in MD simulation execution. So taken together, the observed geometry and frequency of atomic interactions between identified all four small molecules and M^pro^ protein could possibly establish the impact of interactions on binding affinity and hence for exhibiting inhibition or modulation effect of M^pro^ protein biologically.

### Binding free energy using MM-GBSA approach

**Fig. 11:**
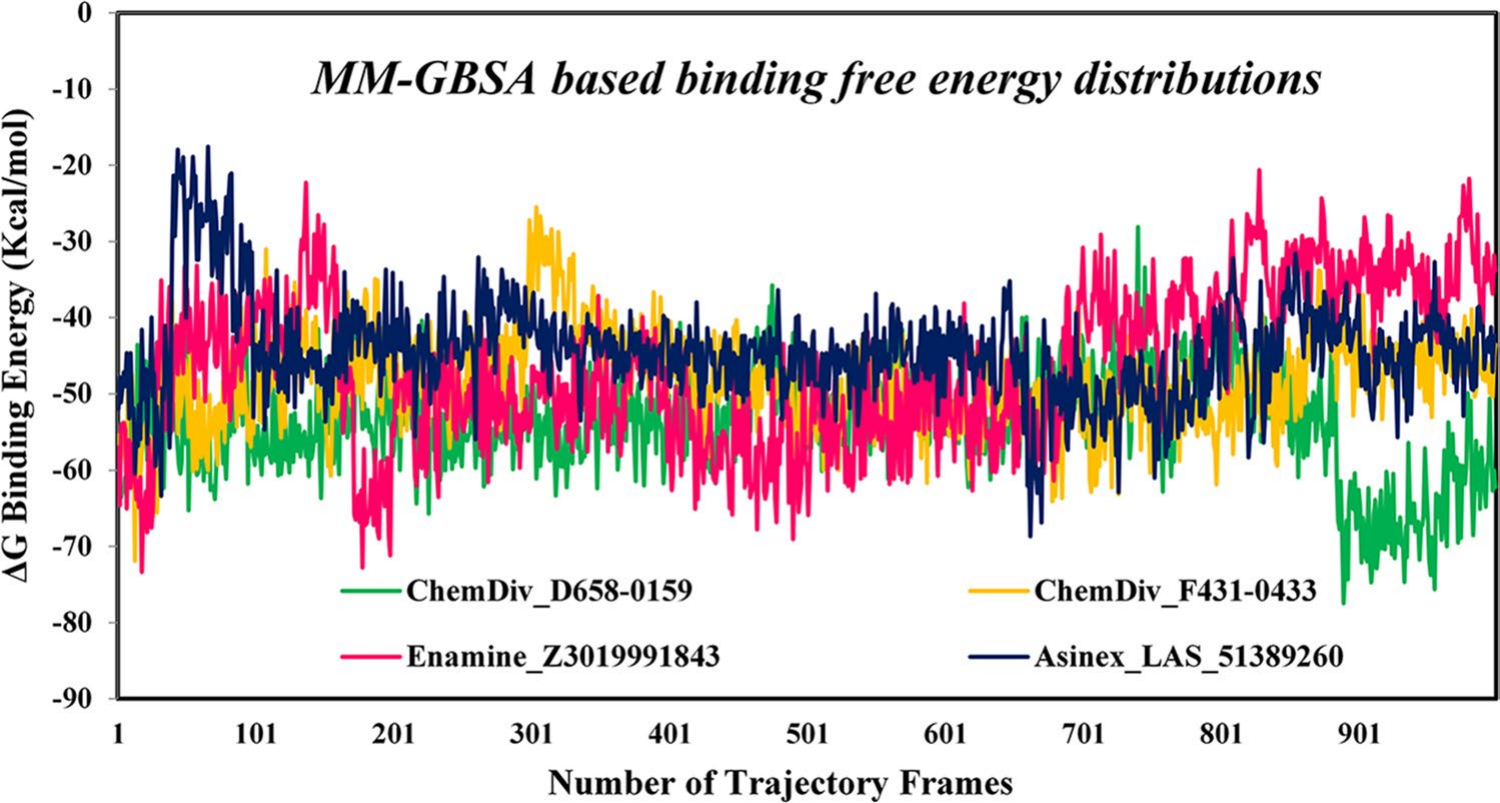
Binding free energy of M^pro^ inhibitors/modulators calculated using MM-GBSA approach

To analyze the binding affinity of the proposed M^pro^ inhibitors, the MM-GBSA approach was used to calculate the binding energy from the entire MD simulation trajectory. The binding free energy of each frame for all molecules was plotted against the frame number, and it is given in Fig. 11. Maximum, minimum and average binding free energy values of ChemDiv_D658-0159, ChemDiv_F431-0433, Enamine_Z3019991843 and Asinex_LAS_51389260 are given in Table 5. Average binding free energy of ChemDiv_D658-0159, ChemDiv_F431-0433, Enamine_Z3019991843 and Asinex_LAS_51389260 was found to be −54.846, −50.170, −49.495 and −49.320 kcal/mol, respectively. The above binding free energy is successfully corroborated with the binding free energy obtained using the Prime-MMGBSA approach (Table 1).

**Table 5:** Binding free energy of M^pro^ inhibitors calculated through MM-GBSA approach. Energy is given in Kcal/mol

Compounds	ChemDiv_D658-0159	ChemDiv_F431-0433	Enamine_Z3019991843	Asinex_LAS_51389260
Minimum	−77.452	−71.866	−73.350	−68.674
Maximum	−28.136	−25.539	−20.652	−17.594
Average	−54.846	−50.170	−49.495	−49.320
Standard deviation	± 6.714	± 6.315	± 10.181	± 6.6174

A number of recent studies have already been performed to estimate the binding free energy through MM-GBSA approach of virtually screened M^pro^ modulators. Proposed M^pro^ molecules reported by Kapusta et al. [45] demonstrated the highest negative MM-GBSA-based binding free energy value of about −60 kcal/mol. Choudhary et al. also screened a few interesting M^pro^ inhibitors and calculated binding free energy using the MM-GBSA approach, and the value was varied in the range of about −50 to −80 kcal/mol. In another study, Ibrahim et al. [46] estimated the binding free energy of four proposed M^pro^ molecules and the value was found to be around −56 to −58 kcal/mol. The above reported estimated binding free energy of M^pro^ inhibitors are also comparable to the binding free energy (Table 5) of ChemDiv_D658-0159, ChemDiv_F431-0433, Enamine_Z3019991843 and Asinex_LAS_51389260, identified in the present study.

## Conclusion

A multistep molecular docking-based virtual screening of three anti-viral specific chemical library databases was performed to find out potential M^pro^ inhibitors. In each step of three-level of molecular docking, the low-potential molecules were wiped out. Finally, based on binding free energy, *in silico* pharmacokinetic and toxicity assessment, four molecules were proposed as potential M^pro^ inhibitors for SARS-CoV-2 inhibition. The binding interaction analysis revealed a number of interesting hydrogen bonds and hydrophobic contacts with of catalytic amino acids. It was also found that all molecules were successfully fitted in all four substrate binding pockets. *In silico* ADME and toxicity data of each molecule were indicated highly absorbable in the GI, soluble in nature, easy to synthesis and non-toxic in nature. The drug-likeness parameters also suggested that all molecules possess lead-like characteristics. The number of parameters from MD simulation study was estimated, and all data indicated the stability of the protein–ligand complex in the dynamic states. High binding free energy calculated from the MD simulation trajectory through the MM-GBSA approach has clearly adjudged the potentiality of the molecules towards M^pro^. However, study limitation can be accounted as non-accessibility of any experimental assay that can provide absolute biological potentiality for the proposed chemical entities as SARS-CoV-2 M^pro^ inhibitors/modulators. Although taken together based on exhaustive computational analyses, it can be proposed that all four chemical entities act as potential M^pro^ inhibitors or modulators for managing COVID-19 situation by taking forward these compounds for expediting drug discovery research against this global pandemic; however, it needs to be subjected for experimental validation.
